# Three Plays with a Purpose

**Published:** 1917-07-28

**Authors:** 


### LESSONS IN PUBLIC MORALS.

### Three Plays with a Purpose.

The whirligig of time has its revenges, and the world of fashionable charity having taken up (for the duration of the war) such matters as child welfare, communal kitchens, and anti-social diseases, or rather permitted itself to discover that these things existed at all, it follows as a necessary corollary that the half-dozen plays on these topics, previously held by an all-wise Censor to be too indecent for public performance, should have been rushed into prominence under the usual august auspices as enforcing a salutary, if unpleasant, lesson in public morals

### Two Plays by Brieux.

Of the three we have had so far, Brieux's "Damaged Goods" is much the most forceful in its special pleading. As a work of art it is negligible. Were it not for the splendid conviction of Mr. Fisher White's declamation as the Doctor it would be unutterably tedious, and as it is, Heaven knows what must be its effect on the guileless multitude who have visited it on the strength of the specious "For Adults Only" that is so prominent a feature of the playbills. The play, as everyone knows, deals with the dangers of untreated syphilis. A young man, on the eve of marriage, contracts the disease, visits a specialist who categorically forbids marriage before the statutory two years have elapsed, and in despair, for the projected alliance has social consequences not readily understandable to an English audience, resorts to quack treatment, and is pronounced cured in the course of a few months.

We next see him happily married, but with an ailing child. The doctor is again called, and pronounces the mother, the child, and the nurse who has suckled it to be all infected by the same deadly virus. The girl's father and mother (he is a Deputy and passes for a social reformer) are naturally horror-struck. "Is there no legal remedy?" they ask. "None." replies the Doctor. The Deputy, who for all his public position is as ignorant of these matters as most people, seeks to know more. It is the reaction of the particular upon the general. "Come to my clinic to-morrow," says the Doctor, "and you shall see what manner of folk are visited by this scourge." And so the Deputy, good easy man, sees with his own eyes some of the consequences of the same youthful follies that he himself has been guilty of—a father pleading for the sanity of his only son, a boy of eighteen; an innocent wife, like his own daughter, infected by a careless husband, and, last of all, the little street-walker, seduced by her employer when little more than a child, and now in a spirit of blind revenge bent on spreading broadcast the contagion she carries. It is all true, terribly true, and the Deputy, shaken to the soul, departs with the firm intention of bringing before the Chamber some repressive legislation which shall prevent the perpetuation of horrors such as these.

That is the story in all its naked brutality, for with Brieux there are no half-tones, and the moral is drawn with relentless certainty.

In his "Three Daughters of M. Dupont" (or of Mr. Smith, as Bernard Shaw aptly suggests) he takes the theme of a household to whom money and getting on are the first considerations. Such young women are often faced only with a choice between the three kinds of lives which fall to the lot of Caroline, Julie, and Angèle respectively: withering on the stalk of the family, throwing her bonnet over the mill, or making a loveless marriage. The play is, if you will, a commentary on the social conditions which make the occurrences of "Damaged Goods" the everyday affairs they are. Caroline is made wretched by the contemptuous, unchivalrous attitude of men to a woman who has failed to marry and is forced by circumstances into the economic field, Julie marries desiring the children which alone would render her lot tolerable, and is denied them by a husband whose circumstances and practical estimate of life made them seem an extravagant folly. Angèle, in escaping the fate of her sisters, falls a victim to the sexual rapaciousness of men. The play closes on no ready-made happy ending. Indeed, how could it? The problems of Mr. Smith and his daughters will not be solved by the end of this generation.

### The Art of Ibsen.

In passing from Brieux to Ibsen we leave the *drame à thèse* for the more rarefied atmosphere of high and almost mystic tragedy. The art of Ibsen is never didactic in the stricter sense; he has no axe to grind, and the moral he points is not always apparent at a first glance. The recurring theme in all his plays is the revolt of an individual (not, mark you, a fine, heroic figure, but a mean, petty creature, very much like you and me, *cher lecteur*) against the stuffiness and convention of everyday life, and their struggles to attain something they call the joy of life. A shabby photographer fooling with rabbits in an attic, in the "Wild Duck"; a middle-aged architect falling from his own tower, with the fatuous idea of showing off to a young lady, in the "Master Builder"; a young woman drinking champagne in a suburban drawing-room, in "Hedda Gabler;" Oswald's desire for Regina's youth and strength in "Ghosts"—these are the symbols he adopts to figure mankind's pitiful struggle against the tyranny of environment. Ibsen in "Ghosts" was but little concerned "to bring home to the world that old-time prophecy 'The sins of the fathers shall be visited upon the children" (as a managerial notice puts it). He is vastly more interested in the emotional reactions aroused in the mind of respectability—Pastor Manders—and in Mrs. Alving by the dissolute life led by her dead husband than in reading so obvious a lesson.

Oswald's torment, his blind search after the "joy of life," his hideous death, serve their purpose, but it is a tragedy whose full significance lies deeper than the surface; it is the fate of Mrs. Alving, not of her son, that must arouse our deepest pity.

That a play such as this should have been banned for twenty-three years affords a striking example of the workings of that mysterious beast, the Censor. Moving it undoubtedly is, outspoken, though never in any way to shock the most fastidious, and if the scene of Oswald's madness be considered a bar to presentation, one can point to the numberless scenes of madness, real and feigned, which our stage has witnessed without affront. The acting at the Kingsway Theatre was competent, if not distinguished—one missed Mr. Leon Quartermaine in the part of Oswald, which he played in J. T. Grein's revival three years ago—but one's appreciation was marred by an unfortunate tendency on the part of the audience to treat the whole thing as low comedy. Incidentally the pathology of G.P.I. seems extraordinarily inaccurate.

